# Pre-stimulus BOLD-network activation modulates EEG spectral activity during working memory retention

**DOI:** 10.3389/fnbeh.2015.00111

**Published:** 2015-05-06

**Authors:** Mara Kottlow, Anthony Schlaepfer, Anja Baenninger, Lars Michels, Daniel Brandeis, Thomas Koenig

**Affiliations:** ^1^Translational Research Center, University Hospital of Psychiatry and Psychotherapy, University Bern Psychiatric Services (UPS)Bern, Switzerland; ^2^Chronobiology and Sleep Research, Institute of Pharmacology and Toxicology, University of ZurichZurich, Switzerland; ^3^Center for Cognition, Learning and Memory, University of BernBern, Switzerland; ^4^Department of Child and Adolescent Psychiatry, University of ZurichZurich, Switzerland; ^5^Institute of Neuroradiology, University Hospital ZurichZurich, Switzerland; ^6^Department of Child and Adolescent Psychiatry and Psychotherapy, Medical Faculty, Central Institute of Mental Health, Mannheim/Heidelberg UniversityMannheim, Germany; ^7^Zurich Center for Integrative Human Physiology, University of ZurichZurich, Switzerland; ^8^Neuroscience Center Zurich, University and ETH ZurichZurich, Switzerland

**Keywords:** state dependency, covariance mapping, working memory, BOLD-ICA, frontal-midline theta, temporally coherent brain networks, pre-stimulus state

## Abstract

Working memory (WM) processes depend on our momentary mental state and therefore exhibit considerable fluctuations. Here, we investigate the interplay of task-preparatory and task-related brain activity as represented by pre-stimulus BOLD-fluctuations and spectral EEG from the retention periods of a visual WM task. Visual WM is used to maintain sensory information in the brain enabling the performance of cognitive operations and is associated with mental health. We tested 22 subjects simultaneously with EEG and fMRI while performing a visuo-verbal Sternberg task with two different loads, allowing for the temporal separation of preparation, encoding, retention and retrieval periods. Four temporally coherent networks (TCNs)—the default mode network (DMN), the dorsal attention, the right and the left WM network—were extracted from the continuous BOLD data by means of a group ICA. Subsequently, the modulatory effect of these networks' pre-stimulus activation upon retention-related EEG activity in the theta, alpha, and beta frequencies was analyzed. The obtained results are informative in the context of state-dependent information processing. We were able to replicate two well-known load-dependent effects: the frontal-midline theta increase during the task and the decrease of pre-stimulus DMN activity. As our main finding, these two measures seem to depend on each other as the significant negative correlations at frontal-midline channels suggested. Thus, suppressed pre-stimulus DMN levels facilitated later task related frontal midline theta increases. In general, based on previous findings that neuronal coupling in different frequency bands may underlie distinct functions in WM retention, our results suggest that processes reflected by spectral oscillations during retention seem not only to be “online” synchronized with activity in different attention-related networks but are also modulated by activity in these networks during preparation intervals.

## Introduction

Resting state brain activity undergoes spontaneous fluctuations, and so does brain activity in response to environmental information. The paradigm of state dependent information processing aims at understanding our experiences and actions not only as a function of a perceived input but as an interaction of an input with the momentary state of the organism in general, and the brain in particular. Thus, dysfunctional responses may be understood as consequences of demand-inadequate pre-states at the moment of perception. Based on that, treatment strategies targeting those pre-states may be developed to enhance functionality.

A particularly important component of brain functionality is working memory (WM). It is a central requirement for many cognitive processes and an indicator of mental health (Klimesch et al., 2010; Van Snellenberg et al., 2015). In addition, the activation of WM related brain functions have well-known physiological EEG signatures that make it possible to quantify their recruitment as a function of pre-stimulus state. The present study aimed at understanding the EEG signatures of the recruitment of working-memory specific and other neuronal resources during a WM task as a function of the brain state preceding stimulus presentation. Thereby, this pre-state was defined by the level of activation of well-known brain networks, measured with the BOLD signal.

Numerous EEG studies on WM have used the Sternberg task (for example Jensen and Tesche, 2002; Leiberg et al., 2006; Michels et al., 2008, 2010, 2012; Maurer et al., 2015), since this task can systematically be configured for different memory loads and is moreover able to temporally separate WM into encoding, retention (maintenance) and recall phases of the WM process. These studies found load-dependent increases of spectral amplitude over broad frequency ranges. In the theta range, increases were mostly observed over medial frontal electrodes with a local maximum at the channel Afz and have been localized to the medial prefrontal cortex and the anterior cingulate cortex, regions forming part of a working memory network (WMN) (Onton et al., 2005; Meltzer et al., 2007; Michels et al., 2008, 2010, 2012; Huang et al., 2013). This frontal-midline theta effects represent sustained increases in theta power during stimulus, retention and probe phases of WM tasks and as such facilitate the maintenance of information in WM through central executive functions (Huang et al., 2013).

Further retention and load effects, however of a more phasic nature (Huang et al., 2013), have been observed in the EEG alpha range primarily over central-posterior sites (Michels et al., 2010) and over right posterior areas (Scheeringa et al., 2009). The local maxima were seen at channel Pz (Michels et al., 2008) and O2, respectively (Michels et al., 2010), and were localized to the right middle occipital gyrus with sLORETA (Michels et al., 2010). The alpha retention effect has been associated with increased demands on storage and inhibition of irrelevant stimuli (Obleser et al., 2012). The alpha load effect correlated in some studies with WM performance (Manza et al., 2014).

Also in the beta range increases with retention and to a smaller degree with load have been observed over occipital sites in a visual verbal Sternberg task (Michels et al., 2010). Thereby, beta WM load-effects seem to represent modality-dependent task relevant stimulus features, as shown by Leiberg et al. (2006) in an auditory WM Sternberg task.

Therefore, with the EEG data of our current study, we sought to replicate these existing findings on EEG load-effects during retention.

For BOLD-data, the literature reports several temporally coherent networks (TCNs; Calhoun et al., 2008) to be associated with processes related to WM (Raichle et al., 2001; Greicius et al., 2003; Dima et al., 2014; Kim, 2015). These networks consist of the task negative default mode network (DMN), and task-positive networks, among others the dorsal attention network (dAN), and the left and the right WM network. Increased connectivity between two nodes of the DMN, the posterior cingulate cortex and the medial prefrontal cortex, has been found to correlate with better WM performance (Hampson et al., 2006). The DMN also showed a load-dependent effect in earlier studies, reflected by a suppression of DMN activity during the preparation for high-load compared with low-load trials (Esposito et al., 2006). The dAN includes the frontal eye field, the superior parietal lobule, the inferior frontal junction, the medial inferior parietal sulcus, the medial dorsal superior frontal gyrus and others (Kim, 2015; Zhang et al., 2015). In contrast to the DMN activating to internally allocated attention, the dAN activates during externally allocated attention. WM processes have been associated with DMN and dAN activity in terms of a double dissociation: while DMN activity is suppressed during task preparation and execution but increases after the task, the opposite holds true for the dAN (Kim, 2015). However, no load-effects associated with the dAN are reported. Further, WM tasks were consistently found to engage a distributed network of areas together forming a WMN. This network contains regions as the dorsolateral prefrontal cortex, the anterior cingulate cortex, a posterior portion of the inferior parietal lobule (Dima et al., 2014; Koshino et al., 2014) and according to some studies the pre-supplementary motor area (Van Snellenberg et al., 2015). The WMN can be further divided into a right and a left lateralized prefronto-parietal network, the lWMN, and the rWMN (Jann et al., 2010; Visintin et al., 2015). The lWMN was found to be activated already in task preparation, whereas both WMNs are active during the execution of WM tasks (Visintin et al., 2015). Right-hemispheric dominance has been associated with verbal WM load (Dima et al., 2014).

When analyzed simultaneously, combined EEG-fMRI studies have shown a link between the TCNs and EEG spectral activity for resting state (Jann et al., 2010) as well as for task conditions (Scheeringa et al., 2009; Michels et al., 2010). During rest as well as during task, the correlations of BOLD and theta power resulted in negative values in areas of the DMN (Meltzer et al., 2007; Scheeringa et al., 2008, 2009; Jann et al., 2010; Michels et al., 2010, 2012). Between load-dependent posterior alpha increases and BOLD, negative correlations during WM retention were found in the parietal-occipital midline (Meltzer et al., 2007) as well as in the primary visual cortex and the middle temporal gyrus (Tuladhar et al., 2007). Resting state beta power showed only positive correlations with BOLD responses during resting state in simultaneous EEG/fMRI studies (Michels et al., 2010), primarily in the middle PFC and other regions of the DMN (Laufs et al., 2003).

None of these studies has investigated the effect of pre-stimulus TCN activation upon EEG signatures of WM specific recruitment of brain resources during retention. Thus, in this study we followed a mainly exploratory approach. Nevertheless, one hypothesis can be derived from the literature: Based on studies showing increased pre-stimulus DMN activity leading to deteriorated task performance (Esposito et al., 2006; Li et al., 2007), and the findings on load-dependent increases in theta-power during WM tasks (Onton et al., 2005; Meltzer et al., 2007; Michels et al., 2008, 2010, 2012; Huang et al., 2013), we expected load-dependent decreases in pre-stimulus DMN activity to be predictive of load-dependent EEG theta power increases during the task.

## Material and methods

### Subjects

Data from a total of 24 subjects measured with simultaneous EEG/fMRI during a visual Sternberg task in two different scanners were taken for the BOLD- independent component analysis (ICA) and EEG analyses. Subjects were recruited via university message boards and had normal or corrected-to-normal vision, met the standard fMRI inclusion criteria and gave their written informed consent for participation in the study. None of the subjects suffered from neurological or psychiatric disorders or used psychoactive medication or drugs. The subjects refrained from caffeine and nicotine for four minimum 2 h before the measurement. The study was approved by the local ethics committee of Bern and Zurich, respectively. For the covariance mapping, two subjects had to be excluded because of residual scan artifacts in the EEG data, resulting in 22 subjects for the covariance mapping analysis (age 27.68 years, SD 7.24; 12 f). Of these subjects, 10 had been tested in a Siemens scanner at the Inselspital Bern, 12 in a Philips scanner at the Psychiatric University Clinic Zurich by using the same measurement parameters (see Sections MRI and EEG Data).

### Task

We used a modified Sternberg visual WM task adapted from Michels et al. (2010) allowing for the temporal separation of preparation, encoding, retention and retrieval periods (Sternberg, 1966). Similar versions have been used by Jensen and Tesche (2002), Michels et al. (2008, 2012) and Maurer et al. (2015). During the task, 64 trials (32 per condition) were presented, each consisting of the sequential presentation of a visual stimulus (duration 2.5 s), a retention period (duration 3.5 s), a visual probe (duration 2.0 s), and a fixation cross (random duration between 1.8 and 2.5 s).

The trials were presented in four large blocks each composed of two high- and two low-load sub-blocks. Each sub-block contained four trials of the same load. Before and after each of the four large blocks, a longer baseline condition of 24.5 s consisting of a fixation cross was presented.

**Figure 1:**
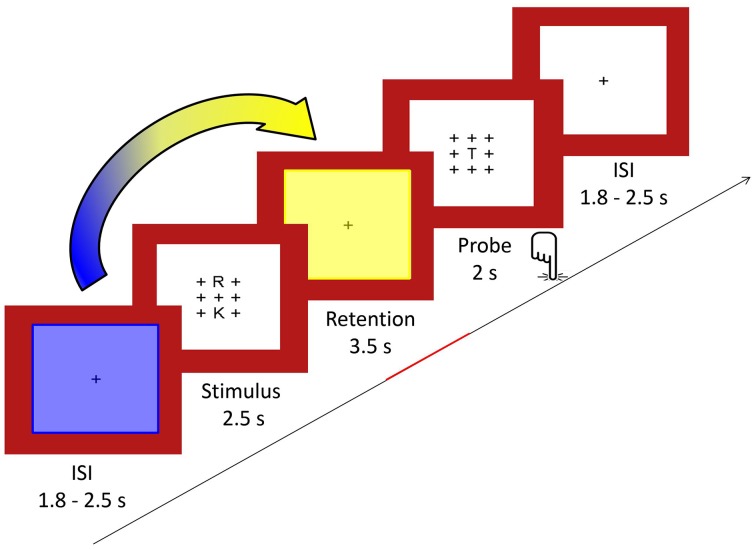
**In the Sternberg WM task, sets of either two or five consonants were presented for 2.5 s (stimulus) and had to be retained in memory for 3.5 s (retention)**. Subsequently, a probe letter was shown for 2 s (retrieval). Subjects indicated by button press whether the probe had been part of the stimulus. As indicated by the arrow, we computed covariance mappings between pre-stimulus percent TCN-activity fluctuations (period marked in blue) and EEG spectral fluctuations from the last 2.5 s of the retention period (period marked in yellow).

For the stimulus, either two (low load) or five (high load) consonants were presented in an array of 3 × 3 items with the remaining positions being plus signs (+). The positions of the consonants excluding the center one were counterbalanced across trials. The stimuli were presented in black font on a white background surrounded by a red frame. Luminance was kept constant. One trial included a stimulus array presented for 2.5 s, followed by the retention period consisting of a centered fixation cross (+) for 3.5 s. The probe consisted of the same array of crosses with the probe letter being presented always in the middle and was presented for 2 s. The probe requested a Yes/No response (forced choice) depending on whether the letter was part of the stimulus set or not. Responses were given with the index and middle finger of the right hand, and response button assignment (“right/left”) was counterbalanced across subjects. The inter-stimulus interval between trials consisted also of a fixation cross and was of a random duration in the range of 1.8–2.5 s (mean 2 s) to minimize preparatory activity. A centered fixation star (^*^) was projected for 24.5 s as a baseline condition five times interspersed. After every block, a short fixation identical to the inter-stimulus interval appeared for 2.5 s. Full task performance summed up to 13 min. A schematic illustration of the task design is given in Figure 1. To become familiar with the task, subjects were given a short practice version of the task outside the scanner.

Subjects viewed the stimuli via goggles (VisualStimDigital MR-compatible video goggles; Resonance Technology Inc., Northridge, CA, USA) while lying inside the scanner. Responses were made via a fiber-optics response button box. In Bern, an in-house MR-compatible response box was used for the task, in Zurich a 4 Button Curve Right Fiber Optic Response Pad (Current Designs, Inc., Philadelphia PA, USA). Stimulus delivery and response registration was controlled by Presentation (Version 170011414, Neurobehavioral Systems Inc., Albany, CA, USA) in Zurich, and by E-Prime (Version 2.0.10.553) in Bern.

Accuracy in terms of percent correct answers and reaction time (RT) was computed from the responses in the low and high WM load conditions, and the differences between the conditions were tested using *t*-tests.

### MRI data

Data were acquired at two different measuring sites and with different 3.0 Tesla (3T) scanners: measuring site (a) was at the University Hospital of Psychiatry in Bern (3T Siemens Magnetom Trio MR Scanner; Siemens 12-channels head coil; Siemens, Erlangen, Germany), measuring site (b) was at the University Hospital of Psychiatry in Zurich (3T Philips Achieva whole-body system; Philips SENSE Head coil 32-elements; Philips Medical Systems, Best, the Netherlands).

The functional T2^*^-weighted MR images were acquired with an echo planar imaging sequence. The characteristics of the sequence were: 250 volumes for the resting state and 406 volumes for the Sternberg WM task, 35 slices, voxel size of 3 × 3 × 3 mm^3^, matrix size 64 × 64, FOV 192 × 192 mm^2^, TR/TE 1960 ms/30 ms. This sequence was optimized for simultaneous EEG/fMRI measurements and hold as similar as possible among measuring sites.

Structural data was acquired with an T1-weighted ADNI-sequence to minimize differences among measuring sites. The ADNI sequence had the following parameters: 176 sagittal slices, slice thickness 1.0 mm, voxel size 1 × 1 × 1 mm^3^, FOV 256 × 256 mm^2^, TR/TE 2300 ms/2.98 ms.

For the preprocessing of the functional MRI data, SPM8 (Welcome Department of Imaging Neuroscience, London, http://www.fil.ion.ucl.ac.uk/spm) was used. After slice timing the data was motion corrected to the mean image. The anatomical T1 was coregistered to the mean image followed by the segmentation into six tissue probability maps. Finally, data was normalized and smoothed using a 6 mm FHWM Gaussian kernel.

#### ICA-approach

BOLD data was parceled into 20 independent components by means of a group ICA implemented in the GIFT toolbox (Calhoun et al., 2008). The independent components were visually compared to networks described in the literature and to templates provided by the GIFT toolbox by means of a spatial similarity tool in order to identify templates for the DMN as a task negative network and the dAN, the lWMN, and the rWMN as task positive networks, driven by our hypothesis introduced above. The four selected templates were back-projected onto the continuous BOLD data of each subject to obtain individual time courses for each TCN and subject. These time courses were deconvolved by the hemodynamic response function and normalized (z-transformed, over time-points). The dynamics of a TCN consist of the percent strength of the respective TCN in the fMRI volume in the middle of each pre-stimulus period (3500 ms before retention onset) in each subject. TCN dynamics were temporally correlated with EEG theta spectral power from the retention intervals (trials of correct responses only). This so-called covariance mapping yielded the spatial distribution of the theta EEG fluctuations during retention associated with the dynamics of the respective TCN in the pre-stimulus interval. In addition, in a time window from −4.3 to 5.5 s in reference to the retention periods, z-transformed TCN dynamics were interpolated on a 0.1 s time-scale and averaged across trials for each subject and load condition. These mean TCN dynamics were then compared moment by moment against zero using two-tailed single one-sample *t*-tests. Similarly, the load conditions were compared using paired *t*-tests. Since the data-window chosen had included a maximum of five MR volumes, these tests were thresholded at a *p*-value<0.01, which corresponds to a Bonferroni-correction with a factor 5.

### EEG data

At both measuring sites, continuous EEG was recorded simultaneously during fMRI-acquisition with a sampling rate of 5 kHz synchronized to the scanner clock and TR to minimize gradient residuals (Mandelkow et al., 2006) using MR-compatible equipment (BrainAmp DC-amplifiers by Brain Products GmbH, Gilching, Germany; EEG caps by EASYCAP GmbH, Herrsching, Germany). Data was recorded with a 0.1 Hz highpass and a 250 Hz lowpass filter and a resolution of 0.5 μV applied to scalp channels. In addition to the scalp channels, an electro-oculogram and an electrocardiogram have been measured. Electrode impedances were kept below 20 kΩ. The electrodes were connected to BrainAmp MR-compatible amplifiers (Brain Products GmbH, Gilching). Fz was used as the recording reference, and the EEG was band-pass filtered at 0.1–250 Hz with a sampling rate of 5 kHz. For patient safety, the EEG amplifiers were battery powered and connected through optical wires to the data acquisition PC.

The Zurich data set has been acquired with 60 scalp channels. A lowpass filter of 1000 Hz and a resolution of 10 μV was applied to the ECG channels. The scalp electrodes covered the 10–20-system plus the channels OI1/2. O1'/2' and Fp1'/2' were placed 15% more laterally to Oz/FPz for a more even coverage. The Bern data set has been acquired using 92 scalp channels at the position of the international 10–10 system.

#### Artifact correction and other preprocessing

For the joint analysis of both data sets, a common electrode subset consisting of 66 channels (AF3, AF4, AFz, C1-6, CP1-6, CPz, Cz, F3-8, Fz, Fc1-6, FCz, Fp1, Fp2, Fpz, FT7-10, Iz, O1, O2, Oz, OI1, OI2, P3-8, Pz, PO1-4, PO7-10, T7, T8, TP7-10) has been created for analyses. A resting state EEG with the eyes closed was recorded outside the scanner and was later used for artifact correction. The start of each volume was automatically marked in the EEG data. The EEG data were preprocessed using Brain Vision Analyzer 2.0.4.368 (Brain Products GmbH, Gilching). The aim of the EEG data preprocessing was to remove scan and artifact and cardio ballistic artifacts (CBA) as well as ocular movement artifacts from the EEG. In a first step, a sliding average MR pulse artifact was computed and subtracted from the individual pulse artifacts for the removal of the scan artifact (Allen et al., 1998). Then, the CBA was removed with a similar averaging method, described in Michels et al. (2010). In a second step, the correction of residual scan and CB artifacts was done with a concatenated ICA procedure previously applied by our group for resting state and task data (Jann et al., 2010; Kottlow et al., 2012): An extended ICA (Bell and Sejnowski, 1995; Lee et al., 1999a,b; Jung et al., 2001) was used to decompose the measured signal into components that appear to be brain activity and components that appear to be artifacts (Jung et al., 2000a,b). To improve the decomposition, the ICA was computed for a dataset that included both the task EEG inside the scanner and the resting EEG outside the scanner, providing a basis for learning the EEG uncontaminated from MR pulse artifacts. To maintain computational feasibility, the EEG was filtered at 1–49 Hz and down-sampled to a sampling rate of 500 Hz. For the objective identification of artifact components, the loadings of all ICA factors were segmented into 2-s epochs and frequency transformed. The average spectral power across epochs of each factor was compared between the measurement outside and inside the scanner. The factors in which the power inside the scanner considerably exceeded the power outside the scanner were identified as MR-related artifacts and removed. Components representing ocular artifacts were identified by their topographic signature. After removal of these specific factors, the EEG obtained inside the scanner was reconstructed from the remaining factors. Still residing artifacts were manually marked, the ECG and EOG channels were removed altogether from the EEG and the data were recomputed to average reference. For all further quantitative analyses the data were down-sampled to 250 Hz and divided into artifact free segments containing the last 2.5 s of the retention period separately for the correctly answered trials of each load condition. From this data, the time-varying EEG frequency spectral amplitude was obtained using a continuous complex Gabor transformation in the frequency range from 2 to 20 Hz, with an envelope that had its half-max at about the latency of a full cycle of the oscillation. These frequency transformed single trial epochs served as basis for all subsequent analyses. For statistics, the values were pooled into three frequency bands, namely Theta (3–7 Hz), Alpha (8–12 Hz), and Beta (13–20 Hz).

#### Replication of the load effect

For the replication of the load-dependent frontal-midline theta effect reported in the literature (Michels et al., 2010), the single epochs of each subject were averaged for each load condition across trials in 5 time bins of 0.5 s duration. In analogy to Michels et al. (2010) a relative load effect computed as the ratio of high load to low load. This relative load effect was compared against the expected constant value of 1.0 using a one-factorial within subject TANOVA for each of the time bin and frequency band. Adjacent time bins where the TANOVA yielded significant effects (*p* < 0.05) were averaged and displayed as t-maps. The same was done in the alpha and beta frequency bands. Since there were a-priori hypotheses about the existence and topographies of load effects, no corrections for multiple testing were applied, but significant results were tested for compatibility with previous studies based on *t*-tests at electrodes reported in these studies.

### Covariance mapping

As schematically shown in Figure 1, we established the predictive value of pre-stimulus TCN activation upon the EEG during the retention period using a so-called covariance mapping, which is based on a linear regression analysis of a predictor (in this case pre-stimulus TCN activation) upon fluctuations of multichannel EEG patterns (in this case the spectral amplitudes during the retention period). Computational details have been described elsewhere (Koenig et al., 2008). Since these covariance maps were established within subjects, we had to test for the stability of the obtained results across subjects. This was done using a permutation test (the so-called topographic consistency test, or TCT) that tests whether in a series of EEG spatial distributions (in our case the individual covariance maps) have something in common that is unlikely to have resulted by chance alone (Koenig and Melie-García, 2010). These TCTs were applied to each TCN, frequency band and load level. In order to minimize the number of tests, we collapsed the data within the entire time period. Since there was no previous study that would provide empirical hypotheses on the outcome of these tests, the resulting *p*-values were thresholded according to a false discovery rate of 5%.

If, within a frequency band, a set of significant TCTs indicated the presence of consistent covariance maps for more than one TCN and/or load-level, we furthermore aimed to clarify if these covariance maps had a spatial distribution that was particular or common for the different TCNs and/or load-levels. This was achieved by computing TANOVAs including those covariance maps that met the above TCT criterion, with TCN and/or load as within subject factors. Significant effects of TCN and/or load-level where further explored by computing t-maps for the relevant contrasts. The labels of the electrodes with the highest/lowest *t*-values were retained and reported to describe the obtained maps, but not used for statistical inference, since this has already been established using the preceding tests.

## Results

### Accuracy

Accuracy as measured by percent correct answers did not differ significantly between the two load conditions (*p* = 0.61). RT was significantly higher for high load compared to low load conditions (*p* < 0.01; mean RT low load 0.82 s (± 0.32), mean RT high load 1.02 s (± 0.38).

### EEG

**Figure 2:**
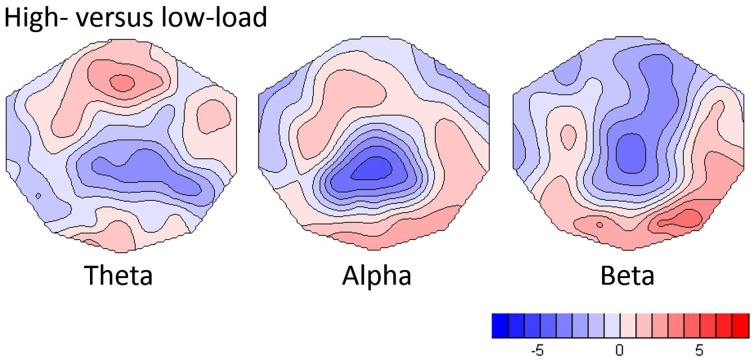
**T-maps showing load-dependent effects for theta, alpha and beta frequency bands**. For the computation, the differences between high- and low-load conditions were normalized by the low load and *t*-tests against one were computed.

As shown in Figure 2, the replication analysis yielded significant load effects in all frequency bands. In the theta band, the TANOVA yielded a significant load effect within the first second of the analysis period (*p* always below 0.05). In the subsequent *post-hoc t*-map analysis in the average of this period, the strongest load effects were seen at frontal-midline electrodes, with a maximal *t*-value at electrode Fz (*t* = 3.44, *p* = 0.0025, df = 21). This findings replicate earlier results for WM retention as described by Michels et al. (2010). The same time windows also displayed significant load effects in the alpha band (TANOVA *p*-values always below 0.01), the corresponding *t*-map was dominated by a midline parietal negativity (*t*-min = −4.98, *p* < 0.0001, df = 21, at electrode Pz) and an occipital positivity which was maximal at electrode O2 (*t* = 2.94, df = 21, *p* = 0.023). In the beta band the first 1.5 s of the analysis period yielded significant TANOVA results, the *t*-map of the contrast computed in this interval showed a negativity with a central parietal midline minimum (*t*-min = −4.90, *p* < 0.0001, df = 21, at CPz) and a right occipital maximum (*t*-max = 4.16, *p* < 0.0005, df = 21, at O2).

### Bold-TCN activity during the pre-stimulus interval

**Figure 3:**
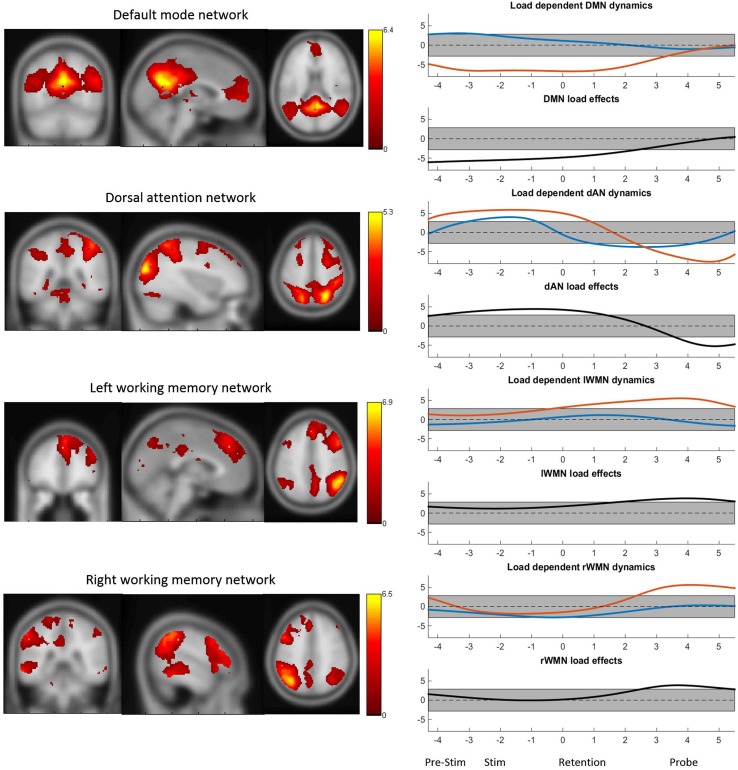
**The four TCNs and their activity over the task for high- and low-load conditions**. The plots on the right show the t-statistics (across subjects) of the z-transformed TCN percent activity over the mean task conditions for low-load (blue) and high-load (red) conditions and *t*-test between high- vs. low-load conditions. Y-axes represent *t*-values and the gray bar represents the significance-threshold. The x-axis represents the time in seconds: −4 to −2.2: pre-stimulus; −2.5 to 0: stimulus; 0 to 3.5: retention; 3.5 to 5.5: probe; 5.5 to 5.8 and −4 to −2.5: pre-stimulus. 0 marks the beginning of the retention period.

The four TCNs as obtained by the group ICA of the continuous BOLD data are shown in Figure 3. The peak MNI coordinates and localized regions of the TCNs can be found in the supplementary material. The most prominent regions however are the bilateral precuneus, the posterior cingulate gyrus and the medial frontal gyrus for the DMN, the superior parietal lobuli, the right middle inferior gyrus including the frontal eye field, the right inferior and superior frontal gyrus for the dAN and the middle frontal gyri including the dorsolateral prefrontal corteces and the pre-supplementary motor areas, the inferior parietal lobuli as well as the anterior cingulate cortex for the WMNs. Additionally, in Figure 3 the TCN activity over the average task trial for both loads is shown, including information when these dynamics were significantly different from the mean. DMN activity was significantly decreased during task preparation (pre-stimulus), encoding (stimulus) and retention in high-load conditions. dAN activity was significantly increased during task-preparation, and decreased during the retention and probe period, these dynamics were significantly larger in the high-load condition. The activity pattern over the task for the lWMN and the rWMN were similar to each other, and showed a significant increase of activation only for the high-load condition during the retention (lWMN only) and probe (lWMN and rWMN).

### Covariance mapping of pre-stimulus TCN activity and retention EEG frequency power

Covariance maps relating the pre-stimulus TCN percent activity fluctuations over trials with the dynamics of EEG spectral wavelet activity of the retention period were computed separately for both WM loads and all four TCNs. This resulted in covariance maps for each of the four pre-stimulus TCNs, each of the three frequency band and both load conditions for each subject (totally 24). To test whether these covariance maps were consistent across subjects, TCTs were calculated for each category with the covariance maps of all subjects, resulting in a total of 24 TCTs. Among these, nine TCTs reached significance when permitting an overall false discovery rate of 5% (theta load 5: DMN, alpha load 2: DMN, dAN, lWMN, rWMN, alpha load 5: dAN, beta load 2: dAN, lMWN, rWMN).

### Tests of the obtained covariance maps for topographic differences as function of TCNs and load levels

**Figure 4:**
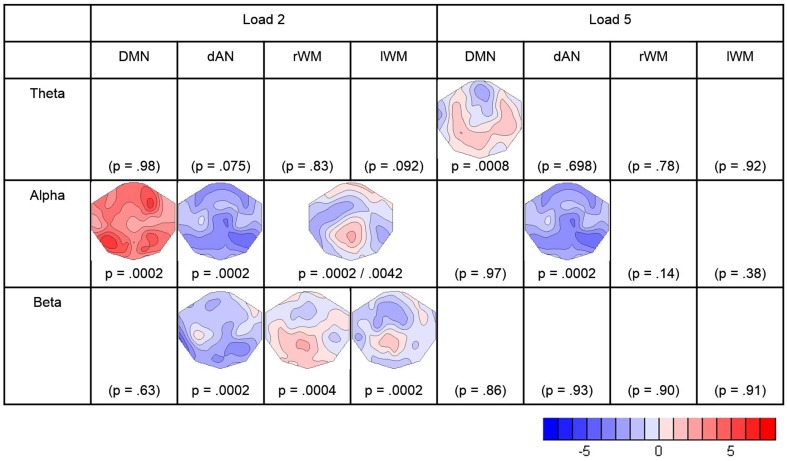
**Result of the TCTs (*p*-values) for each frequency-band and TCN, and t-maps of the significant covariance maps**. Blue indicates negative *t*-values representing negative correlations between the pre-stimulus TCN activity and the retention frequency band power. Red indicates positive *t*-values representing positive correlations. The figure contains the results surviving the adjustment for a FDR of 5%.

In the theta band, the TCT indicated significant covariance maps only for the DMN, and only in the high-load condition. Since in theta band no other TCN showed significant covariance maps, no further analyses were conducted. The t-map (against zero) of the theta-covariance maps of the DMN in the high-load condition is shown in Figure 4, and had a negative maximum at electrode AFz (*t* = −2.701).

In the alpha band, there were significant TCT results for all TCNs in the low-load condition, and for the dAN the high-load condition. To further assess if the covariance maps differed between the four TCNs, a TANOVA across the four covariance maps of the low-load condition was computed. This TANOVA was significant (*p* = 0.0002). Since the covariance maps of the left- and right WMN were visually very similar, we compared them in a separate TANOVA, which was, as expected, not significant (*p* = 0.400). On the other side, the TANOVA comparing the DMN, the dAN, and the combined left- and right- WMN remained significant (*p* = 0.0002) as well as other sub-comparisons (DMN–dAN *p* = 0.0002, DMN–WM *p* = 0.0054, dAN–WM *p* = 0.0096), such that we concluded that the covariance maps of the DMN, the dAN and either WMN with the alpha band can be distinguished, whereas there is no evidence to distinguish the covariance maps of the lWM and the rWM with alpha. In addition, since for the dAN, there were significant covariance maps for the load and high-load conditions, these maps were also compared; the result of this TANOVA indicated no evidence for a difference (*p* = 0.894). Accordingly, t-maps were computed for what the analyses indicated to be the smallest discrete fingerprints of pre-stimulus TCN activation, which were found for the DMN in the low-load condition (with a positive maximum at electrode P7; *t* = 5.85), for the combined left- and right WM also in the low-load condition (positive maximum at Pz, *t* = 2.68), and for the dAN, but combining the low and high load conditions (negative maximum at P8, *t* = −4.76) (Figure 4).

The analysis of the beta-band yielded significant TCT results for the three task-positive networks, but only in the low-load condition. A subsequent TANOVA on these three covariance maps indicated that these maps were significantly different (*p* = 0.0146). The resulting t-maps are again shown in Figure 4.

## Discussion

The present study investigated fMRI network dynamics and EEG spectral changes during the execution of a WM task with two load levels. Specifically, complementing earlier studies reporting load-dependent effects during WM retention in the EEG (Onton et al., 2005; Meltzer et al., 2007; Michels et al., 2008, 2010, 2012; Scheeringa et al., 2009; Huang et al., 2013) and during task preparation in the DMN (Esposito et al., 2006; Manelis and Reder, 2014), we investigated here the relationship among pre-stimulus and task related brain activity.

As a first result, the data replicated a series of findings obtained in similar studies and therefore validates both these results and the findings obtained here. In particular, in the EEG, we found an expected load effect during the retention period in the form of an increase of frontal midline theta in the high-load condition, which is in agreement with a large body of literature linking frontal midline theta to WM, and showing that this link is load dependent (Onton et al., 2005; Meltzer et al., 2007; Michels et al., 2008, 2010, 2012; Scheeringa et al., 2009; Huang et al., 2013).

As also previously reported (Michels et al., 2010), we furthermore found increased alpha and beta power in the high-load condition, maximal at electrode O2. The interpretation of load-related power increases in the alpha range is still under debate: while some argue that this reflects a top-down suppression of irrelevant information during the task (Klimesch et al., 2010; Huang et al., 2013; Roux and Uhlhaas, 2014), others explain this effect with the active participation in attentional processes through the retention of relevant items (Nenert et al., 2012; Manza et al., 2014). Some assign both inhibition of task-irrelevant cortical areas and an active role in conscious attention to the alpha increase during WM (Palva and Palva, 2007). On the other hand, we found load-related alpha and beta decreases at midline centro-parietal electrodes, whereas the paper by Michels et al. (2010) had shown this only for the beta band.

Also the results obtained in the analysis of the event-related dynamics of the TCNs yielded effects that where in agreement with previous studies: DMN activity showed a load-dependent decrease already during task preparation, as previously shown by Manelis and Reder (2014). Beyond that, the mean time courses of the investigated task-positive TCNs over a trial also matched the behavior of these networks described in the literature: the dynamics of the dAN where opposite to the one of the DMN during high-load conditions (Kim, 2015), both WMNs were active during the executing of the WM task, that is the (late) retention and probe phase (Visintin et al., 2015). However, contrary to the findings of Visintin et al. (2015) in our data the lWMN was not yet active in the pre-stimulus period. And both, not only the rWMN (Dima et al., 2014) should a load-effect during the WM task. Further, although many studies reported negative correlations between the DMN and the WMN during resting state and task performance there is evidence that during task preparation both DMN core regions and WMN regions are co-activated (Koshino et al., 2014). This is apparently the case in the low-load conditions of our data.

The principal aim and novelty of our study was the attempt to establish whether and how varying levels of pre-stimulus TCN activation would affect the recruitment of neuronal resources during task execution, as indexed by retention EEG spectral amplitude. The study is thus endorsing the concept of state dependent information processing. This concept assumes that responses to environmental information depend on an interaction of the input with the momentary brain state. Accordingly, part of the variance observed in these responses, and eventually also dysfunctional responses, may be explained by normal variance or abnormal features of the brain state before and at the time of perception.

In our task design, task difficulty was varied pseudo-randomly in blocks of four trials and was thus partly predictable for the subject. Therefore, we could separate anticipatory and random variations of brain state, whereas anticipatory effects were defined as load-dependent mean pre-stimulus effects, while random variations were defined as trial by trial deviations of a TCN from a mean.

As a first result, we found an inverse relation of pre-stimulus DMN activation with frontal midline theta during the retention period, which is in-line with the well-known theta load effect and the findings on pre-stimulus DMN fluctuations indicative of WM functions (Esposito et al., 2006; Hampson et al., 2006; Manelis and Reder, 2014). Previous studies have already established a relation of frontal midline theta and simultaneous DMN activation during the retention period (Michels et al., 2010). Our result extend this finding by showing that DMN activation preceding task execution and quantified prior to any percept to be kept in mind was already predictive for the later recruitment of WM resources. It is thus not that processes related to DMN activation and processes related to WM processes are merely anti-correlated, but that, in the high load condition, pre-stimulus DMN activation “sets the stage” for later WM processes to develop. When task demand was low, these WM processes seemed to be unaffected by pre-stimulus DMN activation.

The predictive power of pre-stimulus DMN upon frontal midline theta during retention in the high load condition supports other findings showing fluctuations of intrinsically organized brain dynamics have effects on cognitive processes and can predict fluctuations in the performance of tasks requiring executive control. A recent study from Nozawa et al. (2014) correlated fluctuations of pre-stimulus TCN activity with reactions times from a color-word Stroop task and was able to show that pre-stimulus DMN fluctuations predicted RT fluctuations, thus constituting “cognitive readiness.” The pre-stimulus TCN-DMN dynamics accordingly may represent a useful measure for anticipatory and preparatory processes, which often cannot be controlled in cognitive experiments. Interestingly, the topography of the pre-stimulus DMN informed theta-band covariance map was also similar to the one found by Jann et al. (2010) through a covariance mapping of resting state DMN with resting state theta power, in the absence of any explicit task. This suggests that frontal midline theta is modulated by DMN activity under very different conditions including rest. Still, the time-lagged relationship between pre-stimulus and task seems not to be a general mechanism, as in our study, the coupling was only significantly present during high-load conditions.

In addition, in the low-load condition, the level of pre-stimulus DMN activation was predictive for alpha increases, which were widespread, but had occipital maxima. As reported in the literature, occipital alpha typically showed positive correlations with DMN activity under resting conditions (Nishida et al., 2015). The result, in conjunction with the finding that the low-load condition on average did not produce a significant suppression of DMN activity, may thus suggest that DMN fluctuations were relatively unaffected by the stimulus and may have extended from the pre-stimulus period into the retention period, such that the encountered alpha band effect in the retention period and pre-stimulus DMN fluctuations represent common, but temporally extended processes.

The task-positive networks had significant covariance maps only in the alpha and beta band. Prestimulus dAN was predictive for a widespread suppression of alpha in the retention period, independent of load level. Since both load-levels also showed an increase of dAN activity before the task, this might indicate that a strong activation of attentional systems prior to stimulus processing might also increase the amount of resources recruited during the retention period, as indicated by alpha-suppression. In addition, in the low-load condition, pre-stimulus activation of left and right WM networks induced an increase of alpha at midline parietal electrodes which was opposite to the alpha band effect of high-load at these electrodes. One may thus speculate that randomly occurring processes engaging WM functions in the pre-stimulus period had a negative impact on task related WM processes during retention.

In the beta band, we found, at least for task-positive networks, a correspondence in significance and topography with the covariance maps of the alpha band. As argued previously (Nishida et al., 2015), in combined EEG-fMRI studies, alpha and low beta-band fMRI correlates were often found to be similar, such that the interpretation of these findings may follow those of the alpha band.

In general, based on previous findings that neuronal coupling in different frequency bands may underlie distinct functions in WM retention (Palva et al., 2010) our results suggest that processes reflected by spectral oscillations during retention seem not only to be momentary EEG counterparts of activity in different attention-related networks but are also affected by activity in these areas during the pre-stimulus or preparation intervals. Thus, the proper functioning of the brain already in the preparatory state before a stimulus seems to be a necessary prerequisite for successful WM processing. Future research will have to further investigate these mechanisms. However, the finding of this interdependence may be important for the development of treatment options in cases of WM deficits.

## Author contributions

MK wrote the manuscript. TK, LM, and DB drafted the work and advised the analyses. MK, AB, AS, DB, and TK drafted the experimental design. MK and LM set up the task design, AS and AB acquired and preprocessed the data. TK, MK, and AS wrote scripts for data analysis. MK and TK analyzed and interpreted the data. DB contributed to the interpretation of the data and revised the work for intellectual content. MK, TK, and AB created the figures. TK revised all versions of the manuscript. MK, AB, AS, LM, DB, and TK approved the final version and agreed on all aspects regarding the submitted work.

### Conflict of interest statement

The authors declare that the research was conducted in the absence of any commercial or financial relationships that could be construed as a potential conflict of interest.

## Abbreviations

CBA: cardio ballistic artifact
dAN: dorsal attention network
DMN: default mode network
ICA: independent component analysis
lWMN: left WMN
RT: reaction time
rWMN: right WMN
TCT: Topographic consistency tests
TCNs: temporally coherent networks
WM: working memory
WMN: working memory network
3T: 3 Tesla.
