# Supplementary material for: Pre-stimulus BOLD-network activation modulates EEG spectral activity during working memory retention
Source: Front Behav Neurosci. 2015 May 6;9:111. doi: 10.3389/fnbeh.2015.00111 (PMC4422031; doi:10.3389/fnbeh.2015.00111)
Supplement: Supplementary file 1 [file Table1.DOCX]

**Supplementary material:**

Table S1: Regions of the identified four TCNs that formed the basis for the subsequent analyses. The extraction of the max values and the labeling of the coordinates was done with the “write Tailairach table” utility included in the gift toolbox. This procedure applies a localization based on the Talairach Daemon. The threshold for the beta-values was set at 3.5, distance between contiguous voxels was 4mm. Only positive regions are listed.

| TCN | Region | Random effects: Max Value (x,y,z) |
| --- | --- | --- |
| DMN | Precuneus | 9.2 (-20, -66, 49)/11.6 (30, -72, 35) |
|  | Superior Parietal Lobule | 9.4 (-24, -55, 60)/11.4 (22, -59, 56) |
|  | Middle Temporal Gyrus | 8.6 (-34, -76, 24)/11.4 (38, -76, 24) |
|  | Superior Occipital Gyrus | 8.9 (-32, -78, 28)/10.8 (38, -76, 28) |
|  | Angular Gyrus | 6.3 (-34, -74, 31)/10.0 (36, -72, 31) |
|  | Middle Occipital Gyrus | 8.9 (-32, -82, 21)/9.3 (34, -81, 21) |
|  | Inferior Parietal Lobule | 6.4 (-34, -50, 56)/8.7 (44, -36, 53) |
|  | Postcentral Gyrus | 5.9 (-18, -51, 63)/8.2 (44, -34, 50) |
|  | Cuneus | 7.6 (-28, -80, 32)/7.5 (30, -80, 32) |
|  | Middle Frontal Gyrus | 4.6 (-26, 3, 55)/6.7 (30, 9, 60) |
|  | Inferior Temporal Gyrus | 6.5 (-53, -64, -2)/5.1 (48, -70, -2) |
|  | Superior Frontal Gyrus | 5.0 (-24, 15, 58)/6.4 (26, 7, 55) |
|  | Inferior Frontal Gyrus | 4.1 (-53, 13, 21)/5.6 (48, 9, 22) |
|  | Paracentral Lobule | 3.7 (0, -38, 50)/5.4 (4, -46, 59) |
|  | Superior Temporal Gyrus | 3.9 (-46, 6, -5)/4.7 (48, -61, 18) |
|  | Fusiform Gyrus | 4.3 (-44, -67, -12)/4.5 (48, -57, -12) |
|  | Precentral Gyrus | 4.5 (-55, -4, 39)/4.0 (59, -17, 40) |
|  | Culmen | 4.4 (-16, -37, -12)/3.8 (2, -45, -8) |
|  | Insula | 4.2 (-42, 4, -4) |
|  | Cingulate Gyrus | 4.0 (6, -37, 41) |
|  | Medial Frontal Gyrus | 3.9 (-20, 3, 51) |
|  | Inferior Occipital Gyrus | 3.9 (-42, -70, -3) |
|  | Thalamus | 3.9 (16, -29, 12) |
| dAN | Precuneus | 8.5 (-20, -66, 49)/11.0 (30, -72, 35) |
|  | Middle Temporal Gyrus | 8.4 (-30, -75, 20)/10.7 (38, -77, 22) |
|  | Superior Occipital Gyrus | 8.0 (-28, -80, 28)/10.4 (38, -76, 26) |
|  | Superior Parietal Lobule | 8.6 (-24, -57, 58)/10.2 (24, -57, 58) |
|  | Angular Gyrus | 5.9 (-34, -76, 31)/9.6 (38, -76, 30) |
|  | Middle Occitipital Gyrus | 8.2 (-32, -79, 21)/7.8 (36, -83, 19) |
|  | Cuneus | 8.0 (-26, -76, 31)/7.9 (32, -80, 33) |
|  | Inferior Parietal Lobule | 6.7 (-30, -50, 54)/7.9 (34, -50, 56) |
|  | Postcentral Gyrus | 4.5 (-6, -53, 65)/7.3 (44, -34, 50) |
|  | Middle Frontal Gyrus | 5.5 (26, 0, 48) |
|  | Inferior Frontal Gyrus | 5.2 (48, 9, 24) |
|  | Superior Frontal Gyrus | 5.1 (26, 7, 55) |
|  | Inferior Temporal Gyrus | 4.5 (-53, -58, -4)/4.9 (51, -61, -9) |
|  | Precentral Gyrus | 3.6 (-53, -2, 41)/4.2 (59, -17, 41) |
|  | Fusiform Gyrus | 4.1 (50, -61, -12) |
|  | Superior Temporal Gyrus | 3.9 (48, -61, 18) |
| rWMN | Inferior Parietal Lobule | 6.3 (-48, -52, 43)/16.6 (46, -54, 47) |
|  | Superior Parietal Lobule | 4.3 (-38, -56, 51)/16.4 (42, -58, 49) |
|  | Supramarginal Gyrus | 4.4 (-46, -51, 36)/14.7 (51, -49, 37) |
|  | Middle Frontal Gyrus | 4.9 (-44, 50, -1)/13.3 (46, 21, 39) |
|  | Angular Gyrus | 3.6 (-48, -55, 36)/12.6 (48, -55, 36) |
|  | Precentral Gyrus | 11.9 (48, 21, 36) |
|  | Superior Frontal Gyrus | 6.1 (-2, 33, 48)/11.7 (36, 22, 49) |
|  | Medial Frontal Gyrus | 7.1 (-2, 35, 42)/10.2 (4, 35, 39) |
|  | Middle Temporal Gyrus | 4.3 (-63, -27, -5)/9.9 (63, -26, -7) |
|  | Postcentral Gyrus | 9.3 (55, -36, 50) |
|  | Inferior Frontal Gyrus | 3.8 (-48, 47, 0)/9.3 (40, 54, 1) |
|  | Precuneus | 3.6 (0, -70, 46)/8.9 (36, -66, 42) |
|  | Superior Temporal Gyrus | 7.9 (48, -48, 21) |
|  | Cingulate Gyrus | 3.6 (-2, -26, 31)/6.9 (10, -45, 37) |
|  | Declive | 6.6 (-10, -79, -20)/-999.0 (0, 0, 0) |
|  | Anterior Cingulate | 5.6 (8, 41, 13) |
|  | Inferior Temporal Gyrus | 4.8 (59, -14, -16) |
|  | Insula | 4.1 (48, -40, 20) |
|  | Uvula | 3.9 (-14, -71, -23) |
|  | Cuneus | 3.7 (10, -66, 31) |
| lWMN | Inferior Parietal Lobule | 14.9 (-38, -62, 44)/7.1 (34, -58, 40) |
|  | Superior Parietal Lobule | 14.6 (-36, -62, 49)/6.3 (40, -58, 51) |
|  | Precuneus | 13.0 (-34, -64, 40)/5.9 (32, -62, 40) |
|  | Inferior Frontal Gyrus | 11.5 (-46, 43, 5)/5.5 (53, 38, 13) |
|  | Angular Gyrus | 11.1 (-46, -56, 36)/5.4 (34, -58, 36) |
|  | Supramarginal Gyrus | 10.7 (-46, -53, 36)/5.1 (38, -49, 37) |
|  | Middle Frontal Gyrus | 10.6 (-46, 44, -4)/5.3 (50, 36, 18) |
|  | Middle Temporal Gyrus | 9.8 (-61, -37, -5)/5.1 (65, -35, -5) |
|  | Superior Temporal Gyrus | 9.1 (-46, -57, 29)/3.9 (63, -2, 7) |
|  | Precentral Gyrus | 8.0 (-44, 17, 34)/3.5 (61, -1, 11) |
|  | Superior Frontal Gyrus | 7.6 (-34, 14, 51) |
|  | Inferior Temporal Gyrus | 7.1 (-51, -53, -11) |
|  | Medial Frontal Gyrus | 6.6 (-4, 29, 41) |
|  | Fusiform Gyrus | 6.4 (-48, -55, -11) |
|  | Uvula | 5.3 (32, -63, -24) |
|  | Cingulate Gyrus | 5.0 (-2, -33, 33)/3.8 (2, -33, 33) |
|  | Postcentral Gyrus | 4.7 (-51, -31, 49)/4.2 (53, -34, 51) |
|  | Superior Occipital Gyrus | 4.5 (-38, -74, 28) |
|  | Culmen | 4.2 (28, -61, -24) |
|  | Cuneus | 4.0 (0, -90, 17) |
|  | Insula | 3.6 (-40, 17, -1) |
